# Correlation between parathyroid adenoma volume and perioperative outcomes in primary hyperparathyroidism: Does the size matter?

**DOI:** 10.1007/s13304-025-02086-4

**Published:** 2025-01-30

**Authors:** Antonio Fiore, Sophie Eschlböck, Céline Carlen, Ioannis I. Lazaridis, Alexandros Lalos, Raoul Droeser, Tarik Delko, Alberto Posabella

**Affiliations:** 1 Department of Visceral Surgery and Medicine, Inselspital, Bern University Hospital, University of Bern, Bern, Switzerland; 2 University Center of Gastrointestinal and Liver Diseases-Clarunis, University of Basel, Basel, Switzerland; 3 Faculty of Medicine, University of Basel, Basel, Switzerland; 4 Department of Surgery, Obesity & Bariatric Surgery Centre, Limmattal Hospital, Zurich-Schlieren, Switzerland; 5 Department of Surgery, Hirslanden Hospital, St. Anna-Strasse 32, 6006 Lucerne, Switzerland

**Keywords:** Primary hyperparathyroidism, Parathyroidectomy, Parathyroid adenoma, Postoperative complications

## Abstract

**Background:**

Primary hyperparathyroidism (PHPT) due to a parathyroid adenoma stands as one of the most prevalent endocrinological disorders, with focused parathyroidectomy being the established therapeutic strategy.

**Aim:**

This study aims to investigate whether the volume of the pathological gland influences perioperative outcomes and postoperative morbidity.

**Methods:**

A retrospective analysis was conducted on data from 141 patients who underwent focused parathyroidectomy for PHPT at the University Hospital of Basel between 2007 and 2022.

**Results:**

A total of 141 patients underwent surgery, with a mean age of 57.2 years and prevalence of women (64.5%).The volume of the lesion was divided into three groups (low < 1 ml, middle 1–1.99 ml, large > 2 ml) based on pathological specimen analysis. Preoperative calcium and parathyroid hormone (PTH) values were significantly higher in the large volume group compared to the low volume group (*p* < 0.05), while phosphate and vitamin D values were significantly lower (*p* < 0.05). A comparison of adenoma volume in symptomatic patients with asymptomatic patients revealed no statistically significant difference (*p* = 0.845) and the volume of the gland of any group did not influence the length of the operation (*p* = 0.173) and the perioperative morbidity (*p* = 0.108).

**Conclusion:**

Compared to a volume of less than 1 ml, a parathyroid gland volume greater than 2 ml was associated with higher preoperative PTH and calcium levels and lower phosphate and vitamin D levels. The volume of the parathyroid gland does not seem to impact the clinical manifestations, or the incidence of perioperative complications.

## Introduction

Primary hyperparathyroidism (PHPT) is an endocrine disorder characterized by excessive secretion of parathyroid hormone (PTH) [1–6]. PHPT is associated with hypercalcemia and mostly caused by a single benign parathyroid adenoma (85% of cases), less frequently by multiple parathyroid gland disease (hyperplasia and/or multiple adenomas, 15% of cases), and in fewer than 1% of cases by parathyroid carcinoma [1–7]. Currently, the majority (> 80%) of patients with PHPT in the USA and Western Europe are considered asymptomatic [1, 3, 4]; conversely, the remaining patients present symptoms affecting various organs and systems, primarily with kidney stones or osteoporotic fractures [8, 9].

While parathyroidectomy is the standard treatment for PHPT recommended for symptomatic patients, surgical intervention is also suggested for asymptomatic patients showing subclinical end-organ manifestations, skeletal or renal, or for those at risk of disease progression [1, 5, 9].

Considering that a solitary adenoma is the most frequent cause of PHPT, a focused surgical approach should always be performed, and preoperative precise localization through radiological imaging is crucial. [10–13]. Nowadays, a preoperative concordant localization of the adenoma through two different radiological examinations, including neck ultrasound, Technetium-99 m-sestamibi SPECT/CT and/or 4-dimensional computerized tomography, is required [14, 15]. Most institutions currently favor a combination of ultrasound and Technetium-99 m-sestamibi SPECT/CT or in case of conflicting results, a completion of investigations with the choline PET/CT scan [15, 16].

To date, researches have been studying the correlation between parathyroid gland volume and preoperative laboratory values, intraoperative complications as well as postoperative outcomes to investigate the impact of adenoma size on perioperative biochemical findings and postoperative outcomes. Previous studies have reported discordant results suggesting that more investigations are required [17–23].

This study aims to assess the correlation between parathyroid adenoma volume and the clinical manifestations, preoperative biochemical findings, perioperative outcomes, and postoperative morbidity in patients undergoing parathyroidectomy for PHPT.

## Methods

### Inclusion criteria

Patients were included if they met the following criteria: age 18 years or older, confirmed diagnosis of PHPT, and underwent parathyroidectomy according to the international guidelines [5] at the Department of Visceral Surgery, University of Basel, between 2007 and 2022.

### Exclusion criteria

Patients who underwent parathyroidectomy for other diagnosis than PHPT were excluded from our study.

### Data and groups stratification

The following variables of 141 patients have been retrospectively collected and analyzed: demographic data, comorbidities, radiological diagnostic examinations, operative details, postoperative morbidity and mortality, as well as pathological data of the pathological gland. To better investigate the role of the size of the adenoma in our population, the pathological specimens were stratified into three different groups based on volume according to the histopathological examination: Group 1: volume less than 1 ml, group 2: volume between 1 and 1.99 ml and group 3: volume greater than 2 ml (≥ 2 ml).

Out of the initial 141 patients suffering from PHPT by parathyroid single adenoma, 9 were excluded from this analysis due to missing postoperative data on the volume of pathological gland. The remaining 132 patients were categorized into 3 groups based on the adenoma volume reported by the histopathological examination, as shown in Table 2.

To determine whether to prioritize the volume or weight of the pathological parathyroid gland for the analysis of the study, the correlation between the two variables was examined, and a positive correlation was identified (*r* = 0.86, *p* < 0.001). Based on this statistical result, volume has been selected as primary parameter for our analysis to investigate the potential impact of the pathological parathyroid gland volume on perioperative outcomes.

### Statistical analysis

Categorical variables were presented as frequencies (numbers and percentages), and continuous variables were expressed as means ± standard deviation. Differences between groups were analyzed using an independent *t* test and one-way ANOVA, with statistical significance set at p value < 0.05. A confidence interval of 95% was utilized, and Pearson correlation coefficients were computed to explore relationships between variables.

### Ethical approval

Approval for this study was obtained from the research ethics committee of Nordwest- and central Switzerland [EZNK: 2023–00426].

## Results

A total of 141 patients who underwent parathyroidectomy for PHPT from 2007 to 2022 were analyzed. The mean age was 57 years (+–15) with a predominance of females in 64.5% of the cases. Comorbidities were present in 109 patients (77.3%) and are listed in Table 1. The most prevalent preoperative comorbidity was hypertension, observed in 60 patients (42.6%), followed by diabetes mellitus in 21 patients (14.9%).

**Table 1:** Baseline demographic and clinical characteristics

	Total of patients (%)
Sex: Female/Male	91 (64.5%) / 50 (35.5%)
Age (mean ± SD)	57.2 (± 15.1)
Pre-operative comorbidities:	109 (77.3%)
Hypertension	60 (42.6%)
Diabetes	21 (14.9%)
Multinodular goitre	12 (8.5%)
Hypothyroidism	5 (3.5%)
Thyroid nodule	2 (1.4%)
Unifocal thyroid autonomy	2 (1.4%)
Hypergonadotropic hypogonadismus	2 (1.4%)
Paraganglioma	1 (0.7%)
Previous bariatric surgery	1 (0.7%)
Previous neck surgery	15 (10.6%)
Preoperative radiological diagnostic for pHPT	
Neck ultrasound (only)	16 (11.3%)
Cholin-PET/CT (only)	9 (6.4%)
Neck Ultrasound and Cholin-PET/CT	3 (2.1%)
Neck Ultrasound and Tc-99 m Sestamibi-SPECT/CT	108 (76.6%)
Neck Ultrasound, Tc-99 m Sestamibi-SPECT/CT and Cholin-PET/CT	0 (0%)
Symptomatic manifestation of PHPT	
Previous Urolithiasis	34 (25.8%)
Previous Fractures	30 (22.7%)
Muscular manifestation	18 (13.6%)
Asymptomatic manifestation of PHPT	
Serum Calcium	50 ( 37.9%)
Skeletal involvement (pathological DXA)	19 (14.4%)
Renal involvement (eGFR < 60 ml/min)	11 (8.3%)
Age < 50	8 (6.1%)

The preoperative radiological localization of the pathological parathyroid gland was mostly performed by the combined use of two different modalities. In particular, 108 patients (76.6%) received both neck ultrasound and Tc-99 m Sestamibi SPECT/CT scan followed by neck ultrasound and choline PET/CT scan in 3 cases (2.1%). In contrast, neck ultrasound alone was conducted in 16 patients (11.3%) as listed in Table 1.

A total of 82 patients (62.1%) were referred to our department due to the symptoms of PHPT, while 50 asymptomatic patients (37.9%) underwent parathyroid surgery following the recommendations outlined in international guidelines [5]. The pathological manifestations of PHPT, across different patient groups, are summarized in Table 1.

**Fig. 1:**
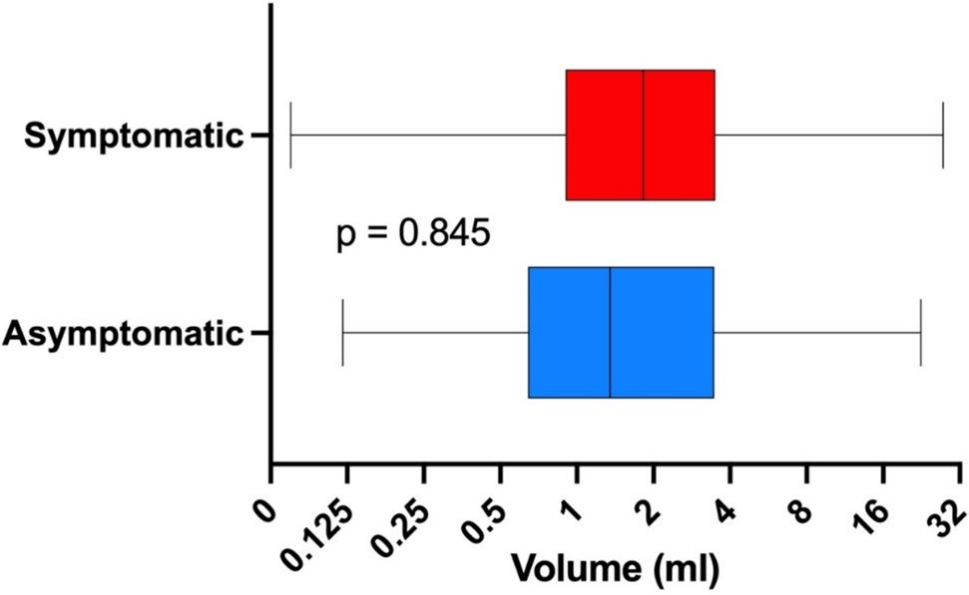
Volume of pathological specimen according to the clinical manifestations of PHPT

A comparison of adenoma volume in symptomatic patients with asymptomatic patients revealed no statistically significant difference (*p* = 0.845), as demonstrated in Fig. 1. A subsequent multivariable analysis among the groups confirmed any statistically significant difference (group 1, *p* = 0.851, group 2, *p* = 0.923 and group 3, *p* = 0.145).

**Fig. 2:**
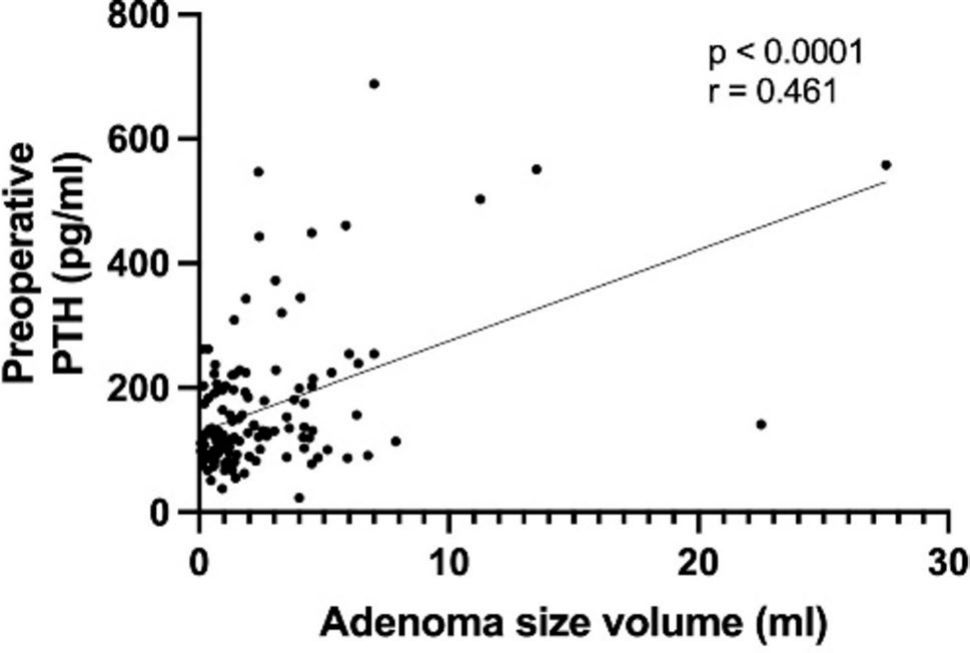
Correlation between the volume of parathyroid adenoma and preoperative PTH

**Fig. 3:**
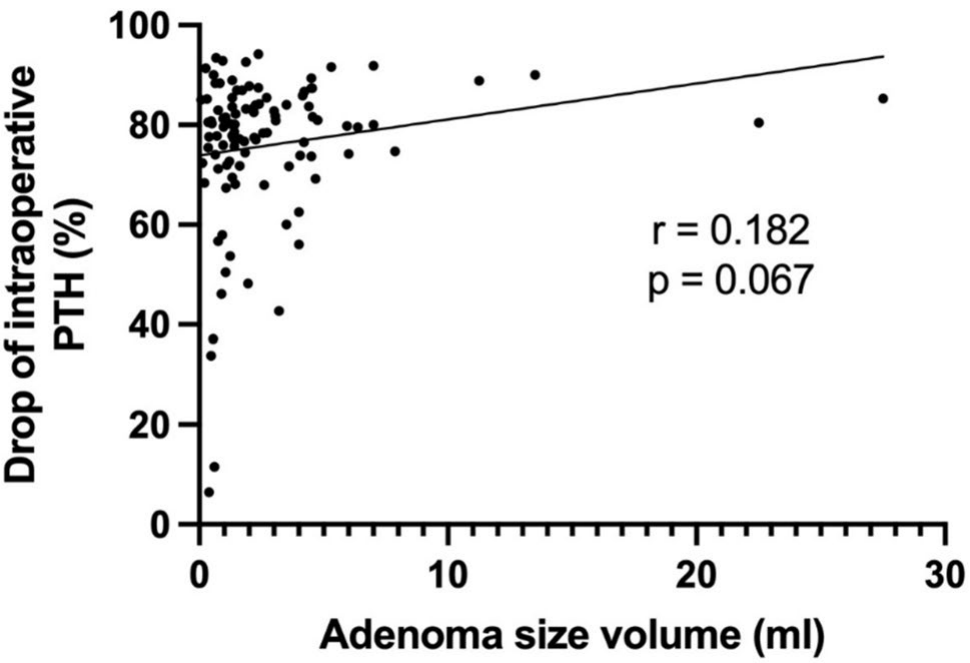
Correlation between the volume of parathyroid adenoma and intraoperative drop of PTH

**Fig. 4:**
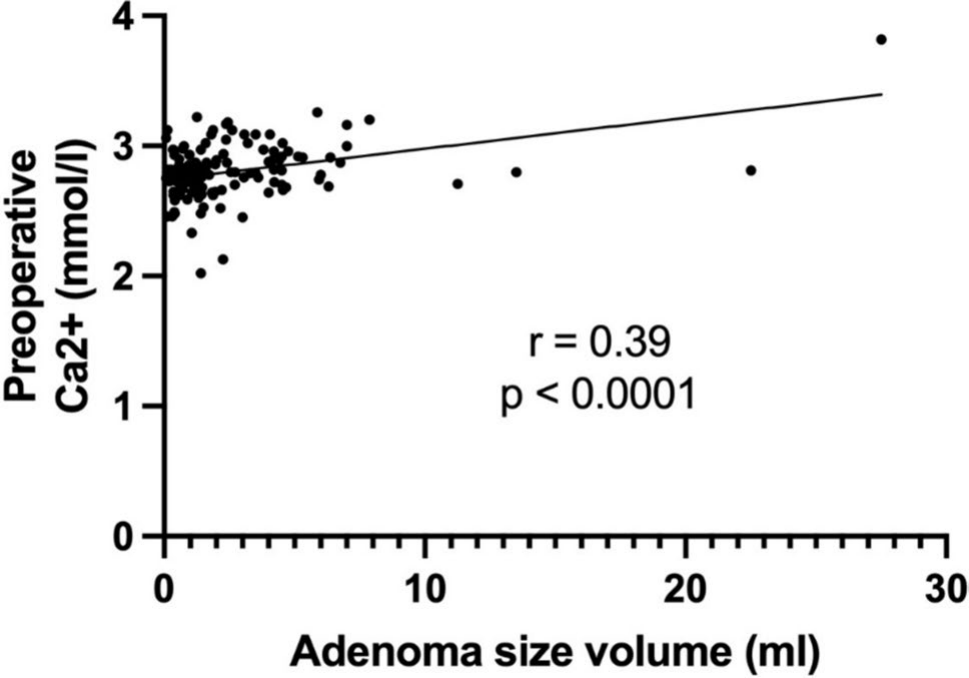
Correlation between the volume of parathyroid adenoma and preoperative Calcium

**Fig. 5:**
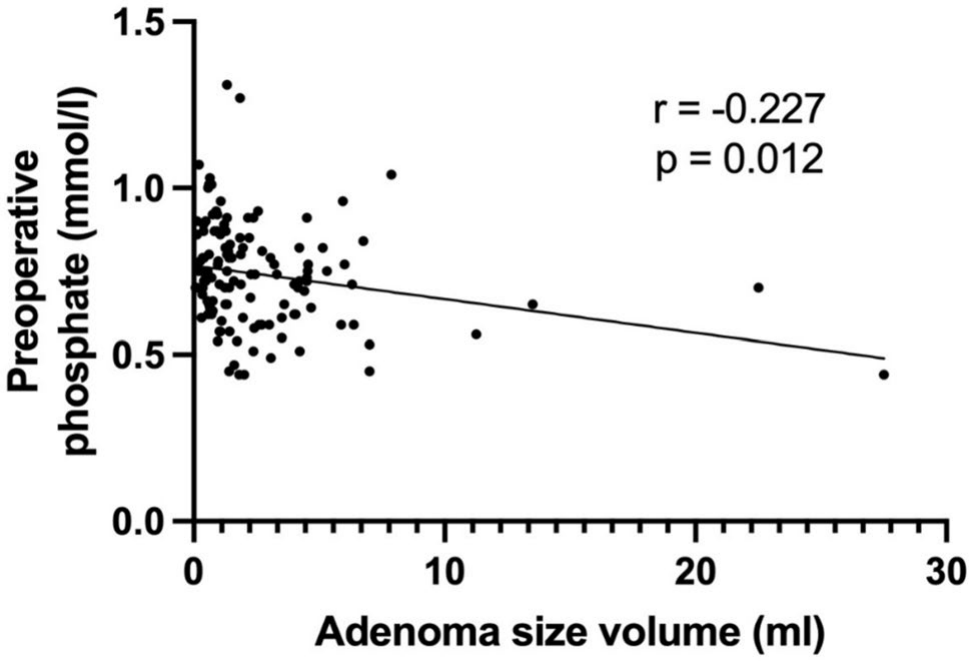
Correlation between the volume of parathyroid adenoma and preoperative phosphate

**Fig. 6:**
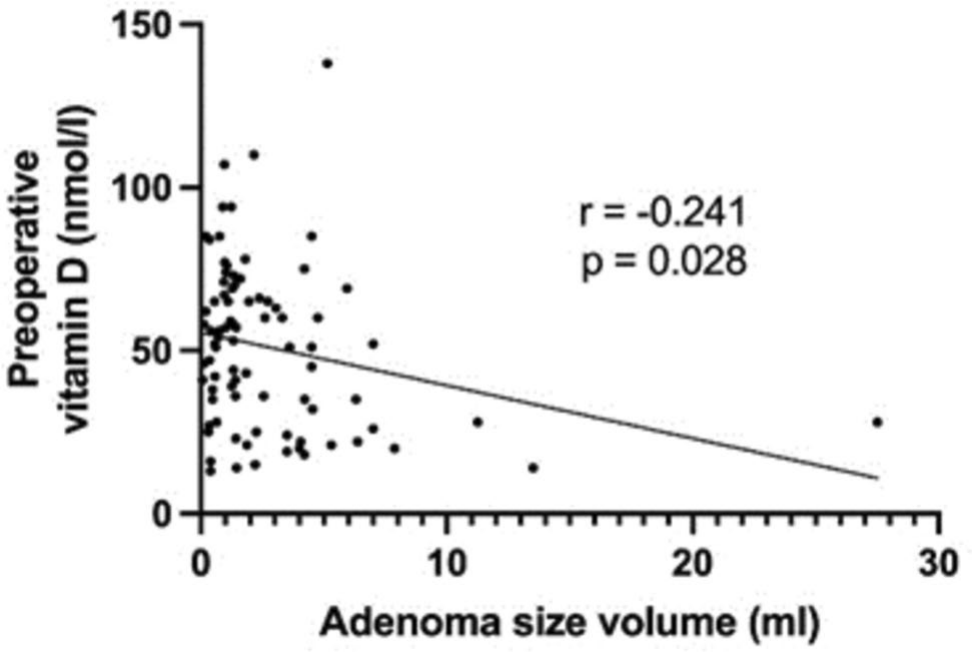
Correlation between the volume of parathyroid adenoma and preoperative vitamin D

A comparison of preoperative laboratory findings among the three groups was conducted and presented in Table 2. Mean preoperative calcium significantly increased with adenoma volume (*p* < 0.05), as well as mean PTH levels (*p* < 0.05). Moreover, mean phosphate and vitamin D levels showed a significant decrease with increasing pathological gland volume (respectively *p* < 0.05). As shown in Figs. 2 and 3, a positive correlation between preoperative PTH levels and adenoma volume (*r* = 0.461, *p* < 0.0001) was seen, while no significant correlation was found between the volume of parathyroid adenoma and the intraoperative drop in PTH levels (*r* = 0.182, *p* = 0.067). Preoperative calcium levels were positively correlated with the adenoma volume (*r* = 0.39, *p* < 0.0001), as illustrated in Fig. 4. Furthermore, preoperative phosphate and vitamin D levels decreased as adenoma volume increased (*r* = −0.227, *p* = 0.012 and *r* = −0.241, *p* = 0.028 respectively), as demonstrated in Figs. 5 and 6. In addition, a comparison of bone mineral density (BMD) T scores across groups revealed no statistically significant differences either preoperatively (*p* = 0.581) or at the 1-year follow-up (*p* = 0.683) (Table 2). Furthermore, no significant correlation was found between parathyroid adenoma volume and preoperative BMD (*r* = 0.188, *p* = 0.099), nor between preoperative parathyroid hormone (PTH) levels and preoperative BMD (*r* = 0.01, *p* = 0.962).

**Table 2:** Pathological manifestation of primary hyperparathyroidism and preoperative data among the groups

	Group 1 (Volume < 1 ml)	Group 2 (Volume 1–1.99 ml)	Group 3 (Volume ≥ 2 ml)	*p*-value
Number of patients	42 (31.8%)	37 (28.1%)	53 (40.1%)	-
Manifestations of PHPT				
Number of patients	26 (61.9%)	25 (67.6%)	31 (58.5%)	0.689
Previous Urolithiasis	9 (21.4%)	11 (29.7%)	14 (26.4%)	0.697
Previous Fractures	12 (28.6%)	10 (27.0%)	8 (15.1%)	0.191
Muscular manifestation	5 (11.9%)	4 (10.8%)	9 (17.0%)	0.692
Asymptomatic patients	16 (38.1%)	12 (32.4%)	22 (41.5%)	0.689
Serum Calcium	4 (9.5%)	5 (13.5%)	10 (18.9%)	0.468
Skeletal involvement (pathological DXA)	4 (9.5%)	3 (8.1%)	4 (7.5%)	0.926
Renal involvement (eGFR < 60 ml/min)	2 (2.7%)	2 (5.4%)	4 (7.5%)	0.903
Age < 50	6 (14.3%)	2 (5.4%)	4 (7.5%)	0.351
Preoperative biochemical markers				
Calcium (mmol/l)	2.8 (± 0.2)	2.7 (± 0.2)	2.9 (± 0.2)	< 0.05
Vitamin D (nmol/l)	54.8 (± 24.4)	55.8 (± 19.8)	45.2 (± 28.7)	< 0.05
PTH (pg/ml)	132.9 (± 57.3)	137.0(± 69.9)	216.3 (± 155.2)	< 0.05
Phosphate (mmol/l)	0.8 (± 0.1)	0.8 (± 0.2)	0.7 (± 0.1)	< 0.05
Preoperative bone mineral density (T-Score)	−1.9 (± 1.1)	−1.9 (± 1.6)	−1.6 (± 1.3)	0.581
Postoperative bone mineral density (T-Score)	−1.8 (± 0.7)	−2.1 (± 1.5)	−1.6 (± 1.5)	0.683
Intraoperative data				
Length of operation (min)	93.7 (± 27)	95.8 (± 27.6)	85.9 (± 24.6)	0.173
Pre-excision PTH (pg/ml)	147.2 (± 88.9)	166.1 (± 55.5)	355.9 (± 636.2)	0.066
Post-excision PTH (pg/ml)*	38.7 (± 29.7)	36.8 (± 16.2)	54.7 (± 53.9)	0.099
Drop of intraoperative PTH (%)**	70.4 (± 22.9)	75.8 (± 10.8)	79.6 (± 9.9)	0.067

^*^ PTH measurement 10 min following complete resection

^**^ Drop of PTH according Miami criterion

The duration of the parathyroidectomy (p = 0.173) and the intraoperative drop of PTH levels (p = 0.067) were not significantly different among the groups (Table 2).

Postoperative complications associated with focused parathyroidectomy are summarized in Table 3. A deep analysis of postoperative complications within 30 days after focused parathyroidectomy stratified by the volume of the parathyroid adenoma revealed that the overall complication rate (group 1 vs 2. OR 0.41, *p* = 0.12; group 1 vs 3. OR 1.2, *p* = 0.23, group 2 vs 3. OR = 0.78, *p* = 0.64) was not different among the groups. Moreover, no difference was seen by stratifying the analysis according to specific complications such as vocal cord palsy (group 1 vs 2. OR 0.25, p = 0.14; group 1 vs 3. OR 1.2, *p* = 0.99, group 2 vs 3. OR = 0.21, *p* = 0.06), hypoparathyroidism (group 1 vs 2. OR 0.62, *p* = 0.70; group 1 vs 3. OR 0.48, *p* = 0.34, group 2 vs 3. OR = 0.77, *p* = 0.76), and hypocalcemia (group 1 vs 2. OR 0.41, *p* = 0.41; group 1 vs 3. OR 0.58, *p* = 0.69, group 2 vs 3. OR = 0.87, *p* = 0.99).

**Table 3:** 30-day complications after parathyroidectomy among the groups

	Group 1 (*n* = 42)	Group 2 (*n* = 37)	Group 3 (*n* = 53)	*p* value
Complications at 30 days n (%)	5 (11.9%)	11 (32%)	14(26.4%)	0.108
Surgical-site infection	0	0	0	–
Bleeding necessitating re-operation	0	0	0	–
Vocal cord palsy	2 (4.7%)	5 (13.5%)	2 (3.7%)	0.198
Hypoparathyroidism	1 (2.3%)	3 (8.1%)	7 (13.2%)	0.146
Hypocalcemia	2 (4.7%)	3 (8.1%)	5 (9.4%)	0.712
Mortality	0	0	0	–

In our study population, neither recurrence nor persistence of PHPT has been observed in the 1-year follow-up.

## Discussion

The results of this study suggested a positive correlation between the volume of the parathyroid adenoma and preoperative PTH and calcium levels, while a negative correlation was seen with phosphate and Vitamin D.

Currently, discordant data regarding the correlation between adenoma volume and preoperative biomarkers are lacking and no definitive consensus has been reached. Several studies support our findings showing a similar correlation between the parathyroid adenoma volume and preoperative hormonal levels [18–22]; conversely, Randhawa et al. did not demonstrate any correlation between the pathological gland volume and preoperative laboratory variables [23].

The possible physiological reason supporting these results has not been well clarified. As Javadov et al. suggested, an increase in parathyroid gland volume is due to a rise in cell numbers, particularly the chief cells. These cells are mainly responsible for PTH secretion, and this may result in higher PTH and calcium levels together with lower phosphate levels [24]. In contrast, other authors have studied the chief cells and oxyphilic cells within parathyroid adenomas from a molecular perspective, without confirming a proportional relationship between the number of chief cells and calcium or PTH levels [25, 26].

Few data are currently available regarding the correlation between the volume of parathyroid adenomas and the clinical and biochemical manifestations of PHPT. A work from Kaszczewska et al. concluded that larger adenomas increase the risk of severe hypercalcemia, suggesting that adenoma size might influence the calcemia [27]. Another study by Gezer et al. pointed out a potential link between the volume of parathyroid adenomas and bone mineral loss at the distal forearm among patients with PHPT, suggesting that larger adenomas may contribute to greater skeletal demineralization in these patients [28].

With the exception of the study by Gezer [28], to our knowledge, no other studies specifically analyzed the relationship between parathyroid adenoma volume and BMD. However, it is well established that PHPT leads to alterations in bone mineralization, resulting in a reduction in BMD [29, 30]. Several studies have also reported that elevated levels of PTH are associated with bone density loss [31, 32]. A study conducted by Rubin et al. observed significant improvements in BMD in patients who underwent parathyroidectomy [33]. In contrast to these findings, our study did not observe any improvement in postoperative BMD at the 1-year follow-up. However, the retrospective design of the study together with the lack of a standardized postoperative protocol for long-term monitoring, including BMD assessment and outpatient follow-ups in cases without recurrence, prevents us from making general conclusions.

On the other hand, the results of this study did not show any significant difference between the volume of the adenoma and the onset of renal, muscular, or bone symptoms, or biochemical altered values (such as GFR or DXA). Concerning urological manifestation of PHPT a Danish study confirms the findings of the present study, showing that adenoma size does not seem to affect the incidence of kidney stones [34].

These findings highlight the complexity of understanding the potential relationship between adenoma volume and the clinical manifestations in patients with PHPT. While some studies have suggested a connection between larger adenomas and specific clinical conditions, such as hypercalcemia or bone mineral loss, others—including ours—have not found any direct association between adenoma size and the clinical manifestations. This underscores the need for further investigation in this area.

In addition, it is important to highlight that in our study cohort, the size of the parathyroid adenoma did not influence the surgical strategy. Our surgical approach consistently involved focused parathyroidectomy, guided by the precise measurement of PTH levels both prior to gland excision and 10–15 min post-excision, in accordance with the established Miami criteria. This method allowed us to ensure the accurate identification and removal of the pathological gland regardless the size. Importantly, unilateral or bilateral neck exploration was not deemed necessary in any of the cases, as our approach relied on preoperative localization studies and intraoperative PTH monitoring to confirm the success of the procedure. Moreover, to further optimize surgical precision and enhance patient safety, intraoperative neuromonitoring was routinely employed during every operation, irrespective of the adenoma’s size. By incorporating these standardized protocols, we were able to streamline the surgical process, minimize operative morbidity, and maintain a high level of confidence in the success of the focused parathyroidectomy approach.

According to Randhawa et al. [23], no correlation between adenoma weight and postoperative hypocalcemia was found. In the present study, the multivariate analysis of the three groups did not show any significant difference between the volume of the parathyroid gland and the onset of perioperative complications, considering particularly the vocal cord palsy or the postoperative hypocalcemia. In contrast, a retrospective national database review performed by Tang et al., which analyzed the rate of morbidity and mortality after parathyroidectomy for PHPT in 14.500 patients, suggested that postoperative complications are positively associated with elevated preoperative PTH and calcium levels [35]. However, specific considerations on parathyroid adenoma volume were not included [35].

Even though there is a paucity of studies specifically investigating the correlation between parathyroid adenoma volume and the incidence of both general and specific complications following parathyroidectomy, our study showed a comparable incidence of postoperative complications as described in the literature [22, 35–37]. Moreover, our results indicate that adenoma volume seems to not be a significant risk factor for the onset of perioperative morbidities. However, our findings must be carefully considered due to the retrospective design, the small population size, and the overall low rate of observed complications.

## Conclusion

Despite the limitation described above, this study shows a small to moderate correlation between parathyroid adenoma volume and preoperative biochemical markers, specifically elevated PTH and calcium levels, along with decreased phosphate and vitamin D levels. Nonetheless, this correlation does not appear to significantly impact the clinical manifestations or the incidence of perioperative complications.

## Data Availability

Not applicable.
